# NANOG Attenuates Hair Follicle-Derived Mesenchymal Stem Cell Senescence by Upregulating PBX1 and Activating AKT Signaling

**DOI:** 10.1155/2019/4286213

**Published:** 2019-12-04

**Authors:** Feilin Liu, Jiahong Shi, Yingyao Zhang, Aobo Lian, Xing Han, Kuiyang Zuo, Mingsheng Liu, Tong Zheng, Fei Zou, Xiaomei Liu, Minghua Jin, Ying Mu, Gang Li, Guanfang Su, Jinyu Liu

**Affiliations:** ^1^Department of Toxicology, School of Public Health, Jilin University, Changchun, China; ^2^Department of Ophthalmology, The Second Hospital of Jilin University, Changchun, China; ^3^Department of Ultrasound, The China-Japan Union Hospital of Jilin University, Changchun, China; ^4^Research Center for Analytical Instrumentation, Institute of Cyber-Systems and Control, State Key Laboratory of Industrial Control Technology, Zhejiang University, Hangzhou, China; ^5^Department of Orthopaedics & Traumatology, Li Ka Shing Institute of Health Sciences, Chinese University of Hong Kong, Prince of Wales Hospital, Shatin, Hong Kong, China

## Abstract

Stem cells derived from elderly donors or harvested by repeated subculture exhibit a marked decrease in proliferative capacity and multipotency, which not only compromises their therapeutic potential but also raises safety concerns for regenerative medicine. NANOG—a well-known core transcription factor—plays an important role in maintaining the self-renewal and pluripotency of stem cells. Unfortunately, the mechanism that NANOG delays mesenchymal stem cell (MSC) senescence is not well-known until now. In our study, we showed that both ectopic NANOG expression and PBX1 overexpression (i) significantly upregulated phosphorylated AKT (p-AKT) and PARP1; (ii) promoted cell proliferation, cell cycle progression, and osteogenesis; (iii) reduced the number of senescence-associated-*β*-galactosidase- (SA-*β*-gal-) positive cells; and (iv) downregulated the expression of p16, p53, and p21. Western blotting and dual-luciferase activity assays showed that ectopic NANOG expression significantly upregulated PBX1 expression and increased *PBX1* promoter activity. In contrast, *PBX1* knockdown by RNA interference in hair follicle- (HF-) derived MSCs that were ectopically expressing NANOG resulted in the significant downregulation of p-AKT and the upregulation of p16 and p21. Moreover, blocking AKT with the PI3K/AKT inhibitor LY294002 or knocking down *AKT* via RNA interference significantly decreased PBX1 expression, while increasing p16 and p21 expression and the number of SA-*β*-gal-positive cells. In conclusion, our findings show that NANOG delays HF-MSC senescence by upregulating PBX1 and activating AKT signaling and that a feedback loop likely exists between PBX1 and AKT signaling.

## 1. Introduction

Mesenchymal stem cells (MSCs)—which are characterized by self-renewal, multipotency, nontumorigenic properties, immune-privilege, and rich sources [1]—are considered to be an ideal resource for stem cell-based regenerative medicine. Increasing numbers of animal tests and clinical trials show that MSCs derived from various tissues, in particular, those from solid tissues [2] (e.g., bone marrow, fat, and hair follicles) and placenta exhibit tremendous therapeutic potential in alleviating cardiac infarction [3], end-stage liver failure [4], and kidney fibrosis [5], as well as in enhancing the recovery of chronic skin ulcers [6] and neuronal regeneration [7]. Hair follicle- (HF-) derived MSCs can be easily derived, highly expanded, and used as one of most popular cell sources for regenerative medicine. HF-MSCs not only take part in the generation of hair follicles but also contribute to hair cycle progression. The senescence of HF-MSCs will not only decrease self-renewal and multipotent capacities but also affect hair growth and regeneration.

NANOG, a homeodomain transcription factor, is expressed in the inner cell mass of embryonic stem cells (ESCs) during early embryonic development and the developing germlines of mammals. In conjunction with Oct4 and Sox2, NANOG plays an important role in maintaining stem cell pluripotency and self-renewal and in reprogramming human somatic cells into pluripotent stem cells [8]. In concert with Oct4, NANOG has been shown to facilitate self-renewal and maintain MSCs in an undifferentiated state by directly binding the *Dnmt1* promoter [9]. Studies by the Andreadis' group showed that the ectopic expression of NANOG delays the culture-associated senescence of hair follicle-derived MSCs, reverses the organismal aging of bone marrow-derived MSCs (BM-MSCs), and restores impaired myogenic potential [2, 10–12]. Furthermore, Munst et al. [13] showed that NANOG enhances proliferation and suppresses replicative senescence in human primary fibroblasts by downregulating p27^KIP1^. These experimental data suggest that the transcription factor NANOG plays a vital role in maintaining self-renewal and in pluripotent or multipotent differentiation in stem cells, while suppressing spontaneous senescence during stem cell culture.

PBX1, a homeodomain transcription factor from the three-amino acid loop extension family, is involved in various developmental processes such as organogenesis and hematopoiesis [14, 15]. In synergy with Hox and Meis proteins, PBX1 targets Fgf10 to regulate the development of the lung mesenchyme [16]. In addition, it actively participates in skeletal muscle differentiation by binding the *Myogenin* promoter [17] and regulates bone mineral density by increasing the expression of *Runx2* and *Osterix*, while decreasing the proliferation of osteoblasts and the formation of bone nodules [18]. Moreover, PBX1 is required for the maintenance and self-renewal of hematopoietic stem cells [15]. Furthermore, PBX1 inhibits the development of adipocytes by promoting the proliferation of adipocyte progenitors postnatally [19]. Studies by Wang and colleagues [20] found an interaction loop between *NANOG* and *PBX1*. By directly binding the proximal promoter of *NANOG*, PBX1 upregulates the expression of endogenous NANOG protein in human ESCs, which may activate downstream cell signaling cascades [21]. Moreover, NANOG has been shown to promote *PBX1* transcription in both human ESCs and NIH3T3 cells [22, 23]. However, the interaction between PBX1 and NANOG during MSC senescence has not yet been studied.

The phosphatidylinositol 3 kinase (PI3K)/protein kinase B (also known as AKT) pathway is considered to be a key pathway in the regulation of cell metabolism-associated processes including proliferation, DNA repair, and senescence [24, 25]. Our previous study showed that EGF promotes the proliferation of human hair follicle-derived mesenchymal stem cells (HF-MSCs) by activating the AKT pathway [26]. Some studies suggest that AKT activation inhibits cellular senescence while reducing the expression of p16, p53, and p21. Further, Palumbo et al. [24] found that macrophage migration inhibitory factor delays human MSC senescence by activating AKT signaling. In addition, Liu et al. [27] showed that activation of the AKT pathway can enhance growth and inhibit senescence in skin-derived precursors. In contrast, Kim et al. [28] revealed that p53-induced cellular senescence is required for cooperation between p21 and AKT. Thus, there is no consistent conclusion regarding the relationship between AKT and cellular senescence. These discrepancies may result from differences in cell types or cell states. Furthermore, the signaling pathways responsible for the delay in HF-MSC senescence as a result of NANOG have not yet been reported.

However, the cellular senescence that results from organismal aging or cell culture exerts considerable effects on the quantity and quality of MSCs, which not only compromises the therapeutic potential of the MSCs but also raises safety concerns for stem cell-based regenerative medicine. As such, it is necessary to develop novel strategies for maintaining MSCs in a highly proliferative but undifferentiated state. To this end, we isolated MSCs from hair follicles and transduced them with lentiviral vectors to express NANOG or PBX1. Our research shows that the ectopic expression of NANOG promotes cell proliferation and delays HF-MSC senescence by enhancing PBX1 expression and activating downstream AKT signaling. Moreover, we uncovered a previously unknown positive feedback loop between PBX1 and AKT in these cells.

## 2. Materials and Methods

The protocols for all experiments in the present study were approved by the Institutional Review Board of the Jilin University School of Public Health.

### 2.1. Isolation, Cultivation, and Phenotypic Characterization of HF-MSCs

The isolation and culture of HF-MSCs were performed according to previously published methods [26, 29, 30]. Briefly, after three washes with phosphate-buffered saline (PBS), hair follicles were transferred into a 24-well culture plate, at one or two follicles per well, and cultured in Dulbecco's Modified Eagle Medium-Ham F-12 (DMEM/F-12; Gibco, Life Technologies, USA) supplemented with 10% fetal bovine serum (FBS; Gibco, USA) and 2 ng/ml of bFGF (Sino Biological Inc., China) at 37°C with 5% CO_2_. The culture medium was changed every 3 days. After spindle-shaped cells migrated out from the hair follicles and reached 80% confluency, cells were digested with 0.25% trypsin and subsequently subcultured (Biosharp, China). Immunofluorescence assays and flow cytometry [26] were then performed to evaluate the expression of the following cell surface markers: CD105, CD90, CD73, CD44, and CD31 (eBioscience, USA).

### 2.2. Lentiviral Plasmid Construction and Transduction

The human *NANOG* coding region (Accession No. NM_024865) was first cloned into the pLVX-IRES-ZsGreen1 lentivirus vector (Youbio, China).

*PBX1* forward (5′-GGAGGGTTTCTCTCCCAACG-3′) and reverse (5′-GGGAGTCTAGGACAGAGGCA-3′) primers were synthesized by Sangon Biotech (Shanghai, China), and *PBX1* cDNA was obtained by PCR amplification (PCR SuperMix Kit; TRANSGEN BIOTECH, China) according to the manufacturer's protocols. After enzymatic digestion and ligation, the human *PBX1* coding region (Accession No. NM_002585) was cloned into the pLVX-IRES-ZsGreen1 lentiviral vector (Youbio, China).

Next, 6 × 10^6^ 293 T cells (stored in the Department of Toxicology, School of Public Health, Jilin University) were seeded in a 100 mm cell culture dish (NEST, China) and cultured at 37°C with 5% CO_2_ in High Glucose DMEM (Gibco, Life Technologies, USA) containing 10% FBS (Gibco, USA) overnight. The following day, 10 *μ*g of lentivirus vector encoding *NANOG* or *PBX1*, 7.5 *μ*g of psPAX2-gag/pol/tat/rev, and 2.5 *μ*g of pMD2.G-VSVG were mixed with 22 *μ*l of EndoFectin™ Max Transfection Reagent (GeneCopoeia, USA) in 1 ml of Opti-MEM (Gibco, Life Technologies, USA) and added onto 293 T cells one drop at a time. After 6 h, the medium was replaced with fresh High Glucose DMEM containing 10% FBS and cultured for another 36 h. At the end of the cultivation period, the supernatant was harvested, filtered through a 0.45 *μ*m polystyrene filter (Millipore, America), and added to HF-MSCs supplemented with 10% FBS, 2 ng/ml bFGF (Sino Biological Inc., China) and 10 *μ*g/ml polybrene (Santa, USA). Subsequently, 72 h after transduction, the HF-MSCs were observed using an inverted fluorescence microscope (Leica DFC500, German) and Western blotting was performed to assess the expression of NANOG or PBX1.

### 2.3. Adipogenesis and Osteogenesis Assays with HF-MSCs

For adipogenic differentiation assays, HF-MSCs ectopically expressing NANOG or overexpressing PBX1, and empty vector (control) were cultured in 24-well plates (in triplicate) for 2 weeks in adipogenic differentiation medium consisting of High Glucose Dulbecco's Modified Eagle Medium (DMEM; Gibco, Life Technologies, USA) with 10% FBS (Gibco, USA), 1 *μ*M dexamethasone (Sigma-Aldrich, USA), 0.5 mM isobutyl-methylxanthine (Sigma-Aldrich, USA), 10 *μ*M insulin (Sigma-Aldrich, USA), and 200 *μ*M indomethacin (Sigma-Aldrich, USA). After the cultivation period, cells were stained with Oil Red O (Sigma-Aldrich, USA) to evaluate the formation of lipid droplets and were subsequently imaged using an inverted fluorescence microscope equipped with a digital camera (Leica DFC500, Germany). To quantify adipogenesis, 200 *μ*l of isopropanol (Beijing Chemical Factory, China) was added to each of the 24 wells to dissolve the Oil Red O at room temperature. After 20 min, OD values were measured at a maximum absorption wavelength of 512 nm using a microplate reader (BioTek, USA), and the concentration of Oil Red O was calculated based on a standard curve that had been created by measuring the optical density of Oil Red O using serial dilutions [31].

For osteogenic differentiation assays, HF-MSCs ectopically expressing NANOG or overexpressing PBX1, and empty vector (control) were cultured in 24-well plates (in triplicate) in osteogenic differentiation medium consisting of High Glucose DMEM (Gibco, Life Technologies, USA) with 10% FBS (Gibco, USA), 0.1 mM dexamethasone (Sigma-Aldrich, USA), 50 mM ascorbate-2-phosphate (Sigma-Aldrich, USA), and 10 nM *β*-glycerophosphate for 4 weeks at 37°C with 5% CO_2_. At the end of the cultivation period, cells were stained with Alizarin Red S (Sigma-Aldrich, USA) to assess the formation of mineralized nodules, and cells were imaged using an inverted fluorescence microscope equipped with a digital camera (Leica DFC500, Germany). To quantify osteogenesis, 200 *μ*l of 10% cetylpyridinium chloride (Sigma-Aldrich, USA) was added to each of the 24 wells to dissolve the calcium salt–Alizarin Red S complex at 37°C for 30 min. The OD values of this complex were measured at a maximum absorption wavelength of 558 nm using a microplate reader, and the concentration of calcium salt–Alizarin Red S complexes was calculated based on a standard curve created by measuring the optical density of a CaCl_2_-Alizarin Red S gradient in 10% cetylpyridinium chloride [31].

### 2.4. Cell Proliferation and Cell Cycle Assays

To assess cell proliferation, HF-MSCs were seeded at 2 × 10^4^ cells per well in a 24-well plate with culture medium containing DMEM/F12 and 10% FBS without bFGF and cultured for 5 days. Cells were washed with PBS, digested with 0.25% trypsin, and counted using a hemocytometer (Qiujing, Shanghai, China) at each time point.

For cell cycle assays, 1 × 10^6^ HF-MSCs transduced with *PBX1* (PBX1 group), *NANOG* (NANOG group), or empty vector (vector group) were harvested and fixed in 70% ice-cold ethanol at −20°C overnight. The following day, HF-MSCs were washed three times with PBS and incubated in 500 *μ*l of propidium iodide with RNase (BD Biosciences, USA) for 15 min at room temperature in the dark, subsequently subjected to flow cytometry (FACSCalibur; BD Biosciences, USA) and analyzed using CellQuest Pro software (BD Biosciences, USA). The cell proliferation index (PI) was calculated as follows: PI = (S + G2/M)/(G0/G1 + S + G2/M) × 100% [26].

### 2.5. Senescence-Associated-*β*-Galactosidase Assay

The detection of cellular senescence was performed by SA-*β*-gal activity assays using a cellular senescence staining kit (Beyotime Biotechnology, China) according to the manufacturer's instructions. After HF-MSCs reached 80% confluency in a 24-well plate, cells were fixed with the fixative from the kit for 15 min at room temperature and washed using PBS. Next, cells were cultured in Staining Solution Mix overnight at 37°C without CO_2_. The following day, cells were washed twice with PBS, and SA-*β*-gal positive cells were observed and calculated based on three randomly selected bright fields (containing at least 200 cells per field).

### 2.6. Western Blotting

For Western blotting, 6 × 10^5^ HF-MSCs were seeded in a 10 mm cell culture dish with DMEM/F12 medium supplemented with 10% FBS and 2 ng/ml bFGF. When cell confluency reached 80%, cells were digested and washed twice with cold PBS. Then, cells were lysed in 250 *μ*l RIPA (Beyotime Biotechnology, China) supplemented with 1% protease inhibitor cocktail (CoWin Biosciences, China) and 1% phosphatase inhibitor cocktail (CoWin Biosciences, China) on ice for 30 min, and subsequently centrifuged at 15,000 g for 20 min at 4°C. Next, the concentration of the supernatant was examined using an Enhanced BCA Protein Assay Kit (Beyotime Biotechnology, China), and 15 *μ*g of protein from each sample was loaded in each well and separated by 10% SDS polyacrylamide gel electrophoresis. Proteins were then transferred from the gels to polyvinylidene difluoride membranes (Millipore, USA), and membranes were incubated in 5% nonfat milk powder (Anchor, New Zealand) with tris-buffered saline containing Tween 20 (TBST) at room temperature for 1 h. Next, blots were incubated with primary antibodies in Primary Antibody Dilution Buffer (Beyotime Biotechnology, China) according to the stated antibody dilution ratio. The primary and secondary antibodies used were as follows: AKT (pan) (C67E7) rabbit mAb (1 : 1000; CST), phospho-AKT (Ser473) rabbit mAb (1 : 1000; CST), NANOG rabbit mAb (1 : 1000; CST), PBX1 rabbit mAb (1 : 1000, CST), p16 rabbit mAb (1 : 1000; ProteinTech Group Inc., USA), p21^Waf1/Cip1^ (12D1) rabbit mAb (1 : 1000; CST), p53 mouse mAb (1 : 1000; Santa Cruz Biotechnology, Inc.), PARP1 rabbit mAb (1 : 1000; Santa Cruz Biotechnology, Inc.), GAPDH mouse mAb (1 : 10000; ProteinTech Group Inc.), HRP-conjugated AffiniPure Goat Anti-Rabbit IgG (H+L) (1 : 5000; ProteinTech Group Inc.), and HRP-conjugated AffiniPure Goat Anti-Mouse IgG (H+L) (1 : 5000; ProteinTech Group Inc.). Images were obtained using a chemiluminescence imaging analysis system (ECL; Tanon 5200; Shanghai Tianneng Technology Co., Ltd., Shanghai, China), and band intensity was analyzed with Tanon Gis analytical software (Shanghai Tianneng Technology Co., Ltd., Shanghai, China).

### 2.7. Dual-Luciferase Reporter Gene Assays

NANOG ectopic expression plasmid- and vector-expressing HF-MSCs were cultured in a 24-well plate and transfected with plasmids that included the *PBX1* promoter pGL3-Basic vector (0.9 *μ*g/well) and pRL-TK vector (0.1 *μ*g/well; Youbio Biological Technology Co., Ltd.). The empty pGL3-Basic vector (0.9 *μ*g/well) and pRL-TK vector (0.1 *μ*g/well) were used as the control conditions. Transfection reagent (1.1 *μ*l) was added to each well (GeneCopoeia, China). After 6 h, the medium was replaced with fresh DMEM/F12 and 10% FBS. The cells were lysed and the supernatant was then harvested, and relative light units (RLUs) were measured after 36 h using the Cytation 3 Cell Imaging Multi-Mode Reader (BioTek Instruments, USA) according to the specifications of the Dual Luciferase Reporter Gene Assay Kit (Beyotime Biotechnology, China).

### 2.8. Inhibition of PI3K/AKT Signaling with an AKT Inhibitor or shRNA

The inhibitor LY294002 (MedChemExpress, USA) was used to block the PI3K/AKT signaling pathway in order to inhibit the phosphorylation of AKT. The optimal treatment concentration and time for LY294002 were determined by Western blotting (data not shown). HF-MSCs that were ectopically expressing NANOG were treated with *PBX1* shRNA (AGCTGTCACTGCTACCAATGT) or *AKT* shRNA (ATCGCTTCTTTGCCGGTAT) to inhibit the expression of PBX1 or AKT, respectively (GeneChem, Shanghai, China). In addition, the appropriate shRNA control was also used. Transfection and verification were performed as previously mentioned in the section of lentiviral plasmid construction and transduction.

### 2.9. Statistical Analyses

Data were statistically analyzed using SPSS version 21.0 (IBM Corp., Armonk, NY, USA). The data are presented as the mean ± standard deviation (SD). Differences between two groups were compared using the independent samples' *t*-test, whereas differences between multiple groups were compared by one-way analysis of variance (one-way ANOVA). *P* < 0.05 was considered to be statistically significant. All results were repeated independently a minimum of three times, except where indicated.

## 3. Results

### 3.1. Isolation and Identification of HF-MSCs

**Figure 1:**
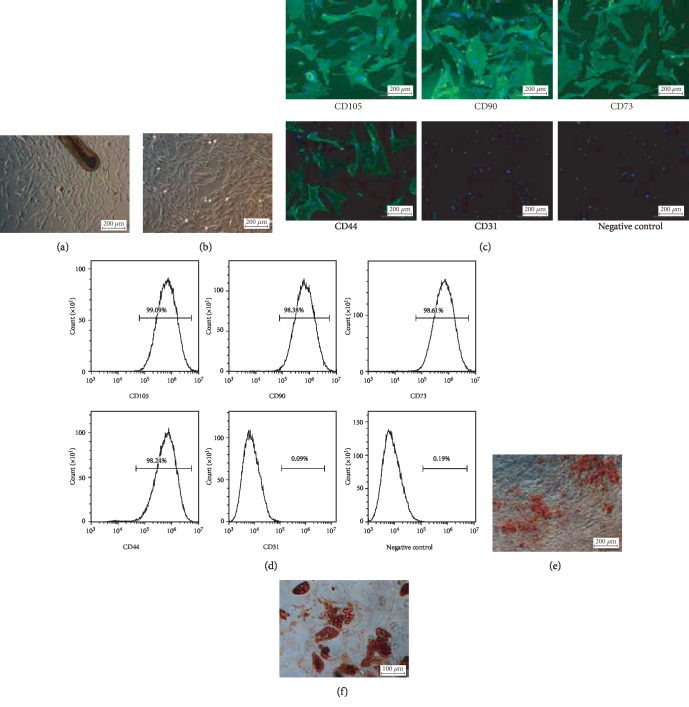
Isolation and identification of hair follicle-derived mesenchymal stem cells (HF-MSCs). (a, b) Cells that migrated from hair follicles exhibited fibroblast-like characteristics. Scale bar = 200 *μ*m. (c) Immunofluorescence assay showing that the cells expressed CD44, CD73, CD90, and CD105 but not CD31. (d) Flow cytometry was used to measure the cell surface expression of MSC markers. (e) HF-MSCs were cultured in osteogenic differentiation media and stained (red) with Alizarin Red S. Scale bar = 200 *μ*m. (f) HF-MSCs were cultured in adipogenic differentiation media and stained (red) with Oil Red O. Scale bar = 100 *μ*m.

HF-MSCs migrated from human hair follicles 10-14 days after seeding in a 24-well plate and displayed a fibroblast-like morphology (Figures 1(a) and 1(b)). Immunofluorescence and flow cytometry assays showed that HF-MSCs expressed CD44, CD73, CD90, and CD105 with low expression of CD31 (Figures 1(c) and 1(d)). Moreover, Oil Red O and Alizarin Red S staining showed that HF-MSCs exhibited osteogenic and adipogenic differentiation potential, respectively (Figures 1(e) and 1(f)).

### 3.2. Ectopic NANOG Expression or PBX1 Overexpression Promotes HF-MSCs Proliferation and Cell Cycle Transition from G0/G1 to S Phase

**Figure 2:**
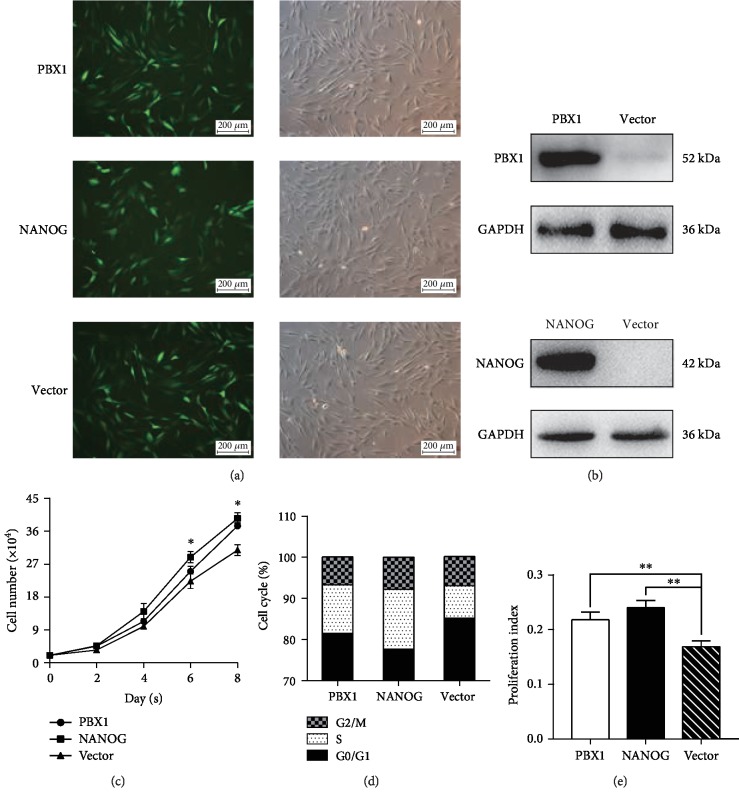
Ectopic NANOG expression and PBX1 overexpression promote proliferation and regulate the cell cycle of hair follicle-derived mesenchymal stem cells (HF-MSCs). (a, b) Images and Western blotting showed ectopic NANOG expression or PBX1 overexpression after lentiviral transduction. Scale bar = 200 *μ*m. (c) Growth curves showed that NANOG and PBX1 promoted proliferation in comparison to that of control (vector only) cells. (d–e) Cell cycle was measured by flow cytometry, which revealed an effect on cell cycle phase distribution. The proliferation indexes of NANOG- and PBX1-expressing cells were higher than that of the control (vector; ^∗^*P* < 0.05, ^∗∗^*P* < 0.01).

Ectopic NANOG expression or PBX1 overexpression via lentivirus transduction was visualized by immunofluorescence imaging and measured by Western blotting (Figures 2(a) and 2(b)). In addition, the results of Western blotting indicated that there was no expression of NANOG and low expression of PBX1 in HF-MSCs (Figure 2(b)).

Cell growth assays showed that the ectopic expression of NANOG or the overexpression of PBX1 significantly increased the quantity of HF-MSCs by 1.4- and 1.2-fold, respectively, in comparison to cells in the vector group (control) on day 8 (Figure 2(c)). Cell cycle assays also showed that the ectopic expression of NANOG or the overexpression of PBX1 also significantly increased the proportion of HF-MSCs in the S phase to 14.56 ± 2.43% in the NANOG group and 11.79 ± 1.84% in the PBX1 group in comparison to that of the vector group (7.87 ± 1.72%). Thus, ectopic NANOG expression or PBX1 overexpression significantly increased the proliferation index by 1.43- and 1.29-fold, respectively, in comparison to that of the vector group (*P* < 0.01; Figures 2(d) and 2(e)).

### 3.3. Ectopic NANOG Expression or PBX1 Overexpression Promotes Osteogenesis, Inhibits Adipogenesis, and Delays Cellular Senescence

**Figure 3:**
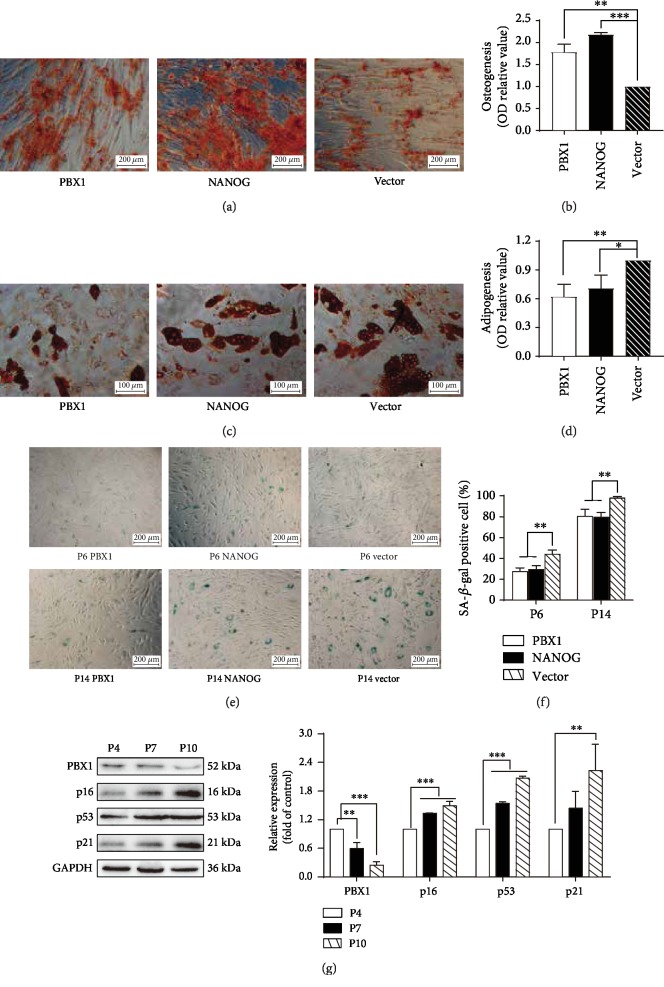
Ectopic NANOG expression and PBX1 overexpression regulate osteogenesis and adipogenesis, respectively. (a, b) NANOG and PBX1 promoted osteogenesis based on Alizarin Red S staining and quantitative detection. Scale bar = 200 *μ*m. (c, d) NANOG and PBX1 repressed adipogenesis based on Oil Red O staining and quantitative detection. Scale bar = 100 *μ*m. (e, f) Blue cells were considered to be positive for senescence based on senescence-associated *β*-galactosidase (SA-*β*-gal) staining. There were fewer positive cells in NANOG- and PBX1-expressing groups in comparison to the vector control group at both passages 6 and 14. Scale bar = 200 *μ*m. (g) Western blotting showed that PBX1 expression decreased and senescence-related protein expression increased with increasing passages (^∗^*P* < 0.05, ^∗∗^*P* < 0.01, ^∗∗∗^*P* < 0.001).

Oil Red O and Alizarin Red S staining showed that ectopic NANOG expression or PBX1 overexpression decreased lipid droplet accumulation by 0.71 ± 0.14- and 0.62 ± 0.13-fold, respectively, in comparison to that of the vector group (*P* < 0.05 and *P* < 0.01, respectively) and also increased calcium nodal formation in HF-MSCs by 2.18 ± 0.05- and 1.78 ± 0.18-fold (*P* < 0.001 and *P* < 0.01), respectively (Figures 3(a)–3(d)). Furthermore, ectopic NANOG expression or PBX1 overexpression significantly decreased the percentage of SA-*β*-gal-positive cells to 28.33 ± 1.73% at passage 6 and 81.33 ± 3.46% at passage 14 (PBX1 vs vector, *P* < 0.01) or 30.33 ± 1.73% at passage 6 and 80.33 ± 2.31% at passage 14 (NANOG vs vector, *P* < 0.01) in comparison to 44.67 ± 2.02% at passage 6 and 98.33 ± 0.67% at passage 14 in the vector group (Figures 3(e) and 3(f)).

### 3.4. Subculture Decreases PBX1 and Increases the Expression of p16, p53, and p21 in HF-MSCs

Western blotting was used to elucidate the relationship between PBX1, cell passage, and proteins related to cell senescence. Our results show that with passage, the relative expression of PBX1 in HF-MSCs was significantly decreased at passage 7 by 0.6 ± 0.13-fold (*P* < 0.01) and at passage 10 by 0.25 ± 0.07-fold (*P* < 0.001) in comparison to that of passage 4. In contrast, the expression of p16, p53, and p21 was significantly increased with passage in HF-MSCs by 1.34 ± 0.01-fold at passage 7 (*P* < 0.001) and 1.49 ± 0.09-fold at passage 10 (*P* < 0.001) for p16, 1.54 ± 0.03-fold at passage 7 (*P* < 0.001) and 2.07 ± 0.04-fold at passage 10 (*P* < 0.001) for p53, and 1.34 ± 0.35-fold at passage 7 (*P* = 0.10) and 2.08 ± 0.54-fold at passage 10 (*P* < 0.01) for p21, in comparison to the expression at passage 4 (Figure 3(g)).

### 3.5. Ectopic NANOG Expression or PBX1 Overexpression Upregulates PARP1 and p-AKT and Downregulates p16, p53, and p21

**Figure 4:**
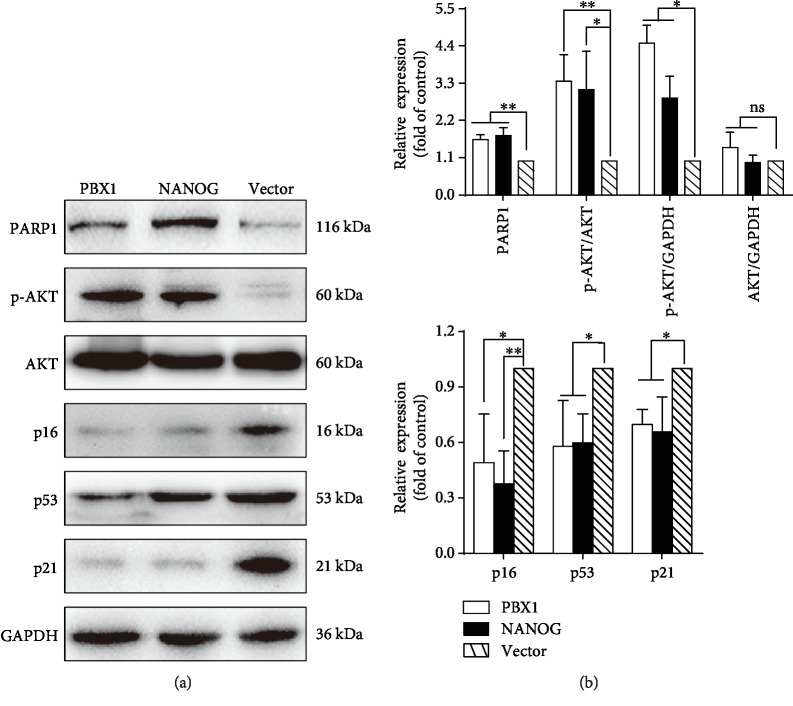
Ectopic NANOG expression and PBX1 overexpression delayed the senescence of hair follicle-derived mesenchymal stem cells (HF-MSCs) via the AKT pathway. (a, b) Western blotting showed that NANOG and PBX1 expression upregulated PARP1 and AKT signaling pathways and downregulated the expression of p16, p53, and p21 in comparison to cells in the vector control group (^∗^*P* < 0.05, ^∗∗^*P* < 0.01).

As AKT signaling is reportedly involved in cell senescence, we aimed to determine if NANOG or PBX1 delays HF-MSC senescence through the activation of this pathway. Indeed, Western blotting showed that NANOG and PBX1 significantly increased PARP1 expression by 1.64 ± 0.09-fold (*P* < 0.01) and 1.75 ± 0.14-fold (*P* < 0.01), respectively, and the expression of p-AKT/AKT by 3.14 ± 0.65-fold (*P* < 0.05) and 3.35 ± 0.46-fold (*P* < 0.01), respectively, in comparison to the empty vector. However, NANOG and PBX1 decreased p16 expression by 0.38 ± 0.18-fold (*P* < 0.01) and 0.49 ± 0.26-fold (*P* < 0.05), respectively, p21 expression by 0.57 ± 0.24-fold (*P* < 0.05) and 0.58 ± 0.24-fold (*P* < 0.05), respectively, and p53 expression by 0.60 ± 0.16-fold (*P* < 0.05) and 0.58 ± 0.25-fold (*P* < 0.05), respectively (Figures 4(a) and 4(b)).

### 3.6. Blocking p-AKT or AKT Knockdown Significantly Downregulates PBX1, Upregulates p16 and p21, and Enhances Cell Senescence

**Figure 5:**
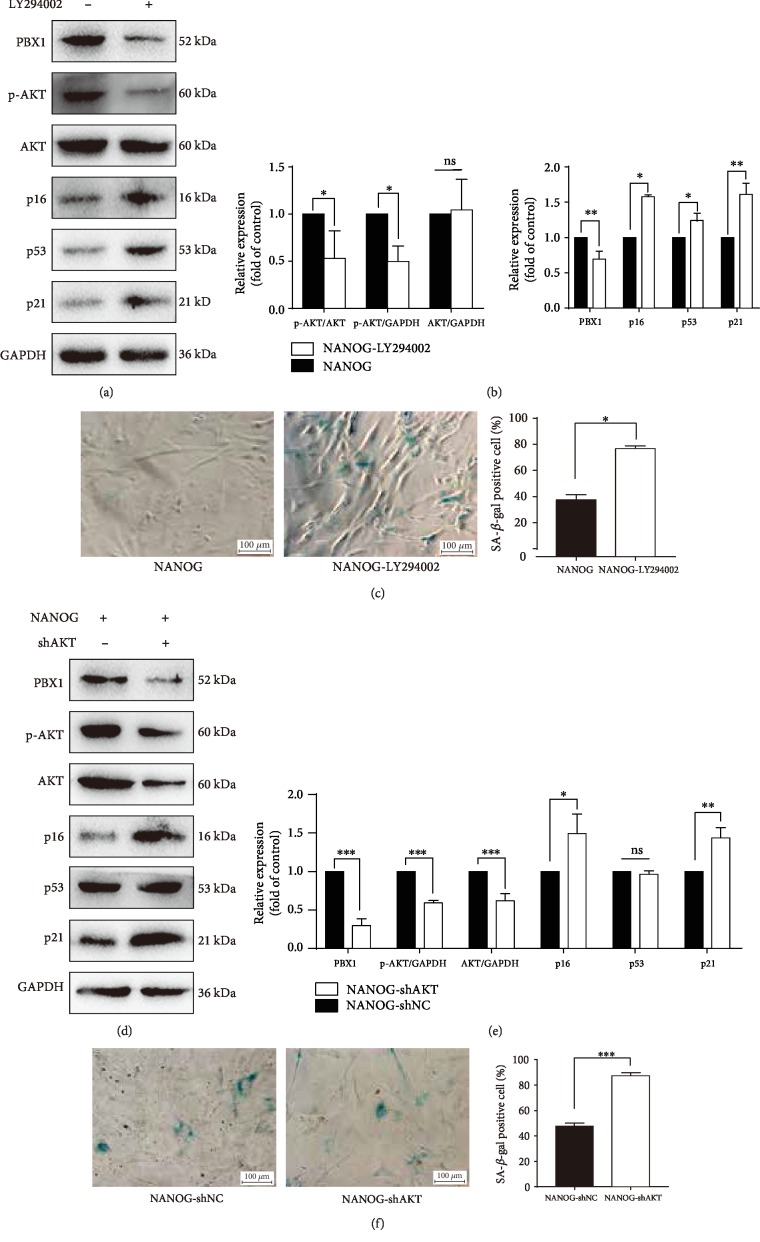
Blocking p-AKT or AKT knockdown significantly downregulates PBX1; upregulates p16, p53, and p21; and enhances cell senescence. (a, b) HF-MSCs that were ectopically expressing NANOG were treated with the PI3K/AKT inhibitor LY294002 (40 *μ*M, 24 h). Western blotting shows that after the inhibition of PI3K/AKT signaling, the expression of p16, p53, p21, PBX1, and p-AKT was significantly altered. (c) Senescence-associated *β*-galactosidase (SA-*β*-gal) staining was used to examine cellular senescence after inhibiting the PI3K/AKT pathway. Scale bar = 100 *μ*m. (d, e) HF-MSCs that were ectopically expressing NANOG were treated with *AKT* shRNA. Western blotting shows that knocking down AKT resulted in the downregulation of PBX1 and an increase in the expression of p16 and p21. Differences in the expression of p16, p21, PBX1, AKT, and p-AKT were statistically significant. (f) SA-*β*-gal staining was used to examine cellular senescence after knocking down *AKT*. Scale bar = 100 *μ*m. (^∗^*P* < 0.05, ^∗∗^*P* < 0.01, ^∗∗∗^*P* < 0.001).

To explore the relationship between PBX1 and p-AKT, p-AKT was blocked using the PI3K/AKT inhibitor LY294002. Western blotting showed that treating HF-MSCs that were ectopically expressing NANOG with this compound significantly reduced PBX1 expression to 69.38 ± 6.62% of control levels (*P* < 0.01) but increased the expression of p16, p53, and p21 to 162.91 ± 19.72% (*P* < 0.05), 123.43 ± 6.25% (*P* < 0.05), and 160.99 ± 9.29% (*P* < 0.01) of control levels, respectively. Furthermore, blocking p-AKT significantly increased SA-*β*-gal-positive cell numbers from 43.33 ± 2.40% to 82.67 ± 1.20% (*P* < 0.05; Figures 5(a)–5(c)). To confirm these results, total AKT was knocked down by RNA interference in HF-MSCs that were ectopically expressing NANOG. Our results showed that the inhibition of AKT significantly reduced PBX1, p-AKT, and total AKT expression to 29.55 ± 5.26% (*P* < 0.001), 59.16 ± 1.86% (*P* < 0.001), and 64.36 ± 4.47% (*P* < 0.001) of control levels, respectively, but increased the expression of p16 and p21 to 149.17 ± 14.76% (*P* < 0.05) and 143.58 ± 7.84% (*P* < 0.01) of control levels, respectively. As expected, total AKT knockdown led to a significant increase in SA-*β*-gal-positive cells from 47.67 ± 1.45% to 87.33 ± 1.45% (*P* < 0.001; Figures 5(d)–5(f)).

### 3.7. Ectopic NANOG Expression Significantly Upregulates PBX1 and Increases PBX1 Promoter Activity

**Figure 6:**
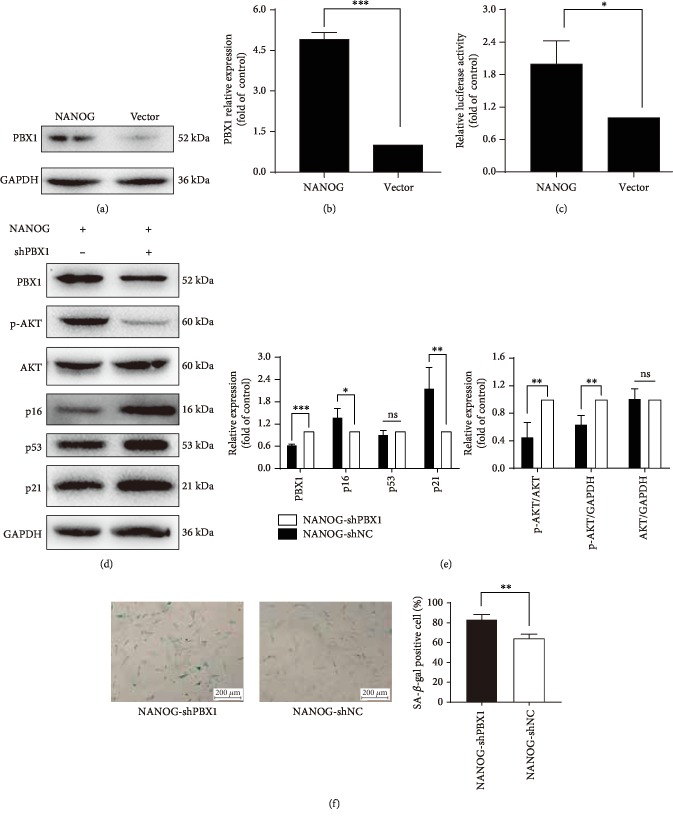
NANOG upregulates the expression of PBX1, while ectopic NANOG expression with *PBX1* shRNA downregulates the phosphorylation of AKT. (a, b) Western blotting shows that ectopic NANOG expression upregulates PBX1 (^∗∗∗^*P* < 0.001). (c) After 36 h of plasmid transfection (*PBX1* promoter pGL3-Basic vector and pRL-TK vector), dual luciferase assays were performed, and luciferase activities (relative light units) were measured in comparison to those of the vector control group (^∗^*P* < 0.05). (d) Hair follicle-derived mesenchymal stem cells (HF-MSCs) ectopically expressing NANOG were treated with *PBX1* shRNA. Western blotting shows that *PBX1* shRNA downregulated p-AKT and increased the expression of p16 and p21. (e) Differences in the expression of p16, p21, and p-AKT, but not p53, were statistically significant. (f) Senescence-associated *β*-galactosidase (SA-*β*-gal) staining was used to examine cellular senescence. Scale bar = 200 *μ*m. (^∗^*P* < 0.05, ^∗∗^*P* < 0.01, ^∗∗∗^*P* < 0.001).

To elucidate the relationship between NANOG and PBX1, Western blotting and dual luciferase reporter assays were performed. Western blotting showed that ectopic NANOG expression significantly upregulated PBX1 expression by 4.11 ± 0.54-fold in comparison to that of the control group (*P* < 0.001; Figures 6(a) and 6(b)). Dual-luciferase reporter gene assays showed that ectopic NANOG expression significantly increased *PBX1* promoter activity by 1.99 ± 0.43-fold in comparison to that of the control group (*P* < 0.05), suggesting that NANOG may increase *PBX1* expression (Figure 6(c)).

### 3.8. Knockdown of PBX1 Downregulates p-AKT, Upregulates p16 and p21, and Enhances Cell Senescence

To further elucidate the relationship between NANOG and PBX1, HF-MSCs that were ectopically expressing NANOG were transduced with *PBX1* shRNA. Western blotting showed that the inhibition of *PBX1* by RNA interference significantly decreased the expression of p-AKT to 52.47 ± 11.13% of control levels (*P* < 0.01) and increased the expression of p16 and p21 to 138.30 ± 13.43% (*P* < 0.05) and 201.21 ± 26.56% (*P* < 0.01) of control levels, respectively (Figures 6(d) and 6(e)). Consistently, *PBX1* knockdown significantly increased the quantity of SA-*β*-gal-positive cells from 64.33 ± 2.33% to 83.67 ± 2.73% (*P* < 0.01; Figure 6(f)).

## 4. Discussion

The hair follicle is an appendage of human skin and contains abundant MSCs. HF-MSCs play crucial roles in the generation of hair follicles and hair cycle progression. HF-MSCs can be used as cell sources for the engineering of vascular tissue and angiogenesis, neural regeneration, and hair follicle reconstruction, and the delivery and controlled release of insulin, resulting in tremendous therapeutic potential in clinical settings [32–35].

As a homeodomain transcription factor, NANOG is crucial for maintaining the self-renewal and pluripotency of ESCs. Our results demonstrated that there was no endogenous expression of NANOG in HF-MSCs in agreement with Shahini et al.'s study [10]. Moreover, there was no endogenous expression of NANOG in BM-MSCs [12]. Not all multipotential stem cells express NANOG based on the above research. Therefore, we did not downregulate the expression of NANOG.

Han et al. [12] suggested that NANOG might prevent senescence in BM-MSCs and improve proliferation and the capacity for myogenic differentiation. In agreement with a study by the Andreadis group, which suggested that ectopic NANOG expression is able to reverse organismal aging in BM-MSCs, our study showed that the ectopic expression of NANOG or the overexpression of PBX1 results in enhanced AKT phosphorylation, increased HF-MSC proliferation, and decreased cellular senescence at early and late passages, followed by a decrease in markers associated with cellular senescence [36], such as p16, p53, and p21. It has been reported that p16^INK4a^—a gene related to antiaging—acts as a cyclin-dependent kinase inhibitor and is crucial for cell-cycle progression and cellular senescence [37]. Although both p27^KIP1^ and p21^CIP/WAF^ are involved in regulating the G1 to S transition, we did not find that NANOG represses cellular senescence by downregulating p27^KIP1^ in HF-MSCs. It has been suggested that this process is likely cell-type specific, which could result in the induction of various patterns of p27^KIP1^ expression. In addition, it has been shown that p21 is downstream of p53 during the regulation of the G1 phase of cell cycle [13]. Furthermore, our results show that NANOG and PBX1 upregulate poly (ADP-ribose) polymerase1 (PARP1)—a key enzyme involved in DNA repair [38]—and attenuate HF-MSC senescence. In agreement with our results, it has been shown that PARP1 activity decreases with age [39] and that a deficiency of PARP1 accelerates aging and reduces the life span of mice [40].

In this study, we found that ectopic NANOG expression or PBX1 overexpression could significantly enhance osteogenesis and decrease adipogenesis, in agreement with previously published studies that show that PBX1 represses adipocyte differentiation by directly regulating PPAR*γ* after the embryonic stage [19], and that NANOG enhances osteogenesis by regulating the BMP pathway [41]. Humans are prone to weight gain and metabolic bone diseases such as osteoporosis as they age [42, 43], and the differentiation potential of BM-SMCs towards an osteogenic lineage decreases or is lost with age [44]. It has also been reported that Sirt3 is involved in age-related adipogenesis and osteoclastogenesis in aging male mice [43]. The same phenomenon of impaired osteogenic and activated adipogenic processes can be observed in aged animals [45]. These data suggest a negative association between adipogenesis and osteogenesis that occurs with aging.

Our results indicate that NANOG and PBX1 are key transcription factors involved in the antiaging process. Moreover, we found that PBX1 expression in HF-MSCs was remarkably decreased with cell subculture, suggesting that it plays a role in maintaining these cells in a proliferative and multipotent state. Most notably, we found that the ectopic expression of NANOG significantly upregulates PBX1 and results in a decrease in the expression of p16 and p21. The upregulation of *PBX1* was further confirmed by dual luciferase assays, as NANOG overexpression significantly increased *PBX1* promoter activity, indicating that it might be upstream of *PBX1*. We performed RNA interference assays to confirm these findings and observed that, as expected, *PBX1* knockdown in HF-MSCs that were ectopically expressing NANOG significantly reduced the expression of p-AKT, increased the expression of p16 and p21, and promoted cellular senescence. These results suggest that NANOG attenuates cellular senescence via the PBX1-mediated regulation of AKT signaling and that PBX1 plays a vital role in attenuating HF-MSC senescence. Indeed, further investigation confirmed that the expression of PBX1 was remarkably decreased over time when subculturing HF-MSCs. A study by Li and Wang [20] showed that both NANOG and PBX1 play crucial roles as transcription factors in maintaining ESCs in a pluripotent state and that there is a positive interaction feedback loop between these makers based on a global sensitivity analysis. However, few studies have reported on the relationship between NANOG and PBX1 in MSCs. Both Boyer et al. [22] and Piestun et al. [23] found that the *PBX1* promoter is a target of NANOG in NIH3T3 and human ESCs based on a genome-scale location analysis. Moreover, both KLF4 and PBX1 have been shown to collaboratively bind the *NANOG* promoter to subsequently regulate gene expression in human ESCs [21]. This was our impetus for exploring the relationship between NANOG and PBX1.

Surprisingly, we found LY294002 increased p53 as well as p16 and p21; AKT knockdown increased only p16 and p21 but p53. These results are consistent with the report of Kim et al.'s group, showing that AKT directly phosphorylates p21^WAF1^ but p53 [46]. This is probably because AKT is an upstream of p21^WAF1^ [47] and LY294002 is a specific inhibitor of PI3K not AKT. Another reason would be that AKT knockdown results in little AKT expression in cellular cytoplasm, then repeated phosphorylation and dephosphorylation by PIP3 or PHLPP1 probably affecting the stability and function of the modified AKT, therefore decreasing the inhibition of p53 [48]. The results of our study also showed that when PBX1 was knocked down after NANOG ectopic expression, the expression of p16 and p21 increased, and p53 expression showed no statistical difference. Accordingly, NANOG could affect AKT phosphorylation, p16 and p21 expression through PBX1, while NANOG did not regulate p53 expression through PBX1. The reason behind this phenomenon is that PBX1 knockdown reduced p-AKT expression, which leads to a similar consequence as AKT knockdown. The result is consistent with our group's research which showed that PBX1 enhances HF-MSCs proliferation by activating AKT and downregulation of p16 and p21 [49]. These results also suggested that there was a feedback loop between PBX1 and AKT signaling.

Although stem cell therapy holds promise for regenerative medicine, stem cells that are derived from aged donors or harvested by repeated subculture tend to lose their capacity for proliferation and multipotent differentiation, which not only compromises their therapeutic efficacy but also raises safety concerns for stem cell therapy. This necessitates the development of strategies for maintaining stem cells in a multipotent and proliferative state. Collectively, our study confirms the presence of a NANOG/PBX1/AKT pathway and a feedback interaction loop between PBX1 and AKT in HF-MSCs. The NANOG/PBX1/AKT pathway plays an important role in attenuating HF-MSC senescence, while the feedback interaction loop between PBX1 and AKT is mutually beneficial for maintaining HF-MSCs in a highly proliferative state with differentiation potential. We believe that our study makes an important contribution to antiaging research and regeneration medicine because there are few previous studies on the role and molecular mechanisms of NANOG in delaying senescence of HF-MSCs.

## 5. Conclusion

This study is the first to report the existence of a NANOG/PBX1/AKT pathway and a feedback interaction loop between PBX1 and AKT in HF-MSCs. The NANOG/PBX1/AKT pathway plays an important role in attenuating HF-MSC senescence, while the feedback loop between PBX1 and AKT is mutually beneficial for maintaining HF-MSCs in a highly proliferative state with the multipotential capacity.

## Data Availability

The data used to support the findings of this study are available from the corresponding author upon request.
